# Predictive Ki-67 Proliferation Index of Cervical Squamous Cell Carcinoma Based on IVIM-DWI Combined with Texture Features

**DOI:** 10.1155/2021/8873065

**Published:** 2021-01-14

**Authors:** Cuiping Li, Mingxue Zheng, Xiaomin Zheng, Xin Fang, Jiangning Dong, Chuanbin Wang, Tingting Wang

**Affiliations:** ^1^Department of Radiology, Anhui Provincial Hospital Affiliated to Anhui Medical University, Hefei 230001, China; ^2^Graduate school, Bengbu Medical College, Bengbu 233030, Anhui Province, China; ^3^Department of Radiation Oncology, Anhui Provincial Hospital Affiliated To Anhui Medical University, Hefei 230001, China; ^4^Department of Radiology, First Affiliated Hospital, University of Science and Technology of China, Anhui Provincial Cancer Hospital, Hefei 230031, China

## Abstract

**Purpose:**

This study aims to determine whether IVIM-DWI combined with texture features based on preoperative IVIM-DWI could be used to predict the Ki-67 PI, which is a widely used cell proliferation biomarker in CSCC.

**Methods:**

A total of 70 patients were included. Among these patients, 16 patients were divided into the Ki-67 PI <50% group and 54 patients were divided into the Ki-67 PI ≥50% group based on the retrospective surgical evaluation. All patients were examined using a 3.0T MRI unit with one standard protocol, including an IVIM-DWI sequence with 10 *b* values (0–1,500 sec/mm^2^). The maximum level of CSCC with a *b* value of 800 sec/mm^2^ was selected. The parameters (diffusion coefficient (*D*), microvascular volume fraction (*f*), and pseudodiffusion coefficient (*D*^*∗*^)) were calculated with the ADW 4.6 workstation, and the texture features based on IVIM-DWI were measured using GE AK quantitative texture analysis software. The texture features included the first order, GLCM, GLSZM, GLRLM, and wavelet transform features. The differences in IVIM-DWI parameters and texture features between the two groups were compared, and the ROC curve was performed for parameters with group differences, and in combination.

**Results:**

The *D* value in the Ki-67 PI ≥50% group was lower than that in the Ki-67 PI <50% group (*P* < 0.05). A total of 1,050 texture features were obtained using AK software. Through univariate logistic regression, mPMR feature selection, and multivariate logistic regression, three texture features were obtained: wavelet_HHL_GLRLM_ LRHGLE, lbp_3D_k_ firstorder_IR, and wavelet_HLH_GLCM_IMC1. The AUC of the prediction model based on the three texture features was 0.816, and the combined *D* value and three texture features was 0.834.

**Conclusions:**

Texture analysis on IVIM-DWI and its parameters was helpful for predicting Ki-67 PI and may provide a noninvasive method to investigate important imaging biomarkers for CSCC.

## 1. Introduction

Cervical cancer is the most frequent malignant tumors in the female genital system worldwide, and cervical squamous cell carcinoma (CSCC) is the most common histological type. In general, cancer is prior to the elderly, but cervical cancer mainly affects young women, most of whom are diagnosed between 35 and 50 years old [1]. Some previous studies have shown that Ki-67 proliferation index (Ki-67 PI) is positively correlated with tumor size, invasion, cancer stage, and patient survival [2]. Another study indicated that the pathological grades of tumors and the Ki-67 PI are critical clinical indicators in the diagnosis and treatment of tumors [3]. However, the Ki-67 PI must be obtained by immunohistochemical staining after the operation. Biopsy remains as the gold standard of diagnosis and can be applied to study molecular markers. However, due to the limited tumor size of biopsy samples, these cannot represent the whole information of tumor heterogeneity, which increases the risk of underestimating the most aggressive components of tumors. Therefore, the importance of using noninvasive imaging techniques to adequately capture the entire tumor characteristic has been recognized a long time ago [4]. The present study explores the value of texture analysis (TA) combined with intravoxel incoherent motion diffusion weighted imaging (IVIM-DWI), in order to predict the Ki-67 PI in CSCC before the operation and to provide a valuable imaging marker for clinical diagnosis and treatment.

## 2. Materials and Methods

### 2.1. Patients

The present retrospective study was approved by the institutional review board, and the need for an informed patient consent was waived. Between September 2018 and March 2020, 285 patients histologically diagnosed at biopsy with cervical malignancies underwent preoperative 3.0T MRI and subsequently received mastectomy. In order to eliminate the effects of the MRI parameter and cervical cancer histologic type on the results, the investigators only included patients with CSCC scanned on the same MRI platform with a unified imaging protocol (*n* = 70). The investigators excluded the following patients: (1) MRI contraindications or MRI quality that cannot meet the diagnostic requirements, (2) patients treated with neoadjuvant chemotherapy, and (3) non-CSCC patients with pathological results. Hence, 70 female patients (mean age, 50.03 ± 9.02 years old; range, 27–76 years old) were included. The process of patient selection is illustrated in Figure S1.

### 2.2. Imaging Protocol

All patients underwent conventional pelvic MRI and axial pelvic IVIM-DWI before the operation. Pelvic MRI was performed using a unit system (GE Signa HDXT 3.0T MRI scanner, GE Healthcare, USA) equipped with an 8-channel phased-array body coil. All patients received an intramuscular injection of 15 mg hyoscine butylbromide at 30 minutes before the MRI scan to prevent gastrointestinal motility. The bladder was kept approximately half-filled, in order to improve lesion visibility without changing the anatomy. Patients were placed in the supine position and were breathing freely during the acquisition.

The scanning range was from the aortic bifurcation to the inferior margin of the pubic symphysis. The scanning parameters were as follows: axial fast spin-echo (FSE) T1-weighted images (T1WI) (repetition time (TR)/echo time (TE): 550/13 msec, NEX: 2, slice thickness/gap: 4 mm/1 mm) and oblique axial and sagittal fat suppression (FS) FSE T2-weighted images (T2WI) (TR/TE: 4,600/30 msec, NEX: 2, slice thickness/gap: 6 mm/2 mm). Axial IVIM-DWI with FS was obtained in the short-time inversion recovery (STIR) sequence using single-shot echo-planar imaging (EPI) pulse sequence with 10 b values (0, 10, 20, 50, 100, 200, 400, 800, 1,200, and 1,500 s/mm^2^), TR/TE: 4,000/65 msec, NEX: 6).

### 2.3. Image Analysis

Two radiologists with more than 10 years of experience analyzed the images without knowing the pathological results of these patients and finally reached a consensus. Using the GE ADW 4.6 postprocessing workstation, the IVIM-DWI images of the largest tumor layer with *b* = 800 s/mm^2^ were analyzed, and the parameters were calculated. The measurement was repeated for three times, and the average value was obtained. When sketching for the region of interest (ROI), the T_2_WI and dynamic contrast-enhanced (DCE) images were referenced to determine the tumor boundary, and the mucus, necrosis, cystic change, and bleeding areas were avoided. The parameters measured by IVIM-DWI included the following: diffusion coefficient (*D*), microvascular volume fraction (*f*), and pseudodiffusion coefficient (*D*^*∗*^).

The IVIM-DWI images with a *b* value of 800 s/mm^2^ were imported into AK (Analysis Kit, Kinetics Version 2.1, GE Healthcare) software (Table S1). The ROIs were determined after the discussion of two senior attending radiologists, and the tumor boundary was manually sketched. Meanwhile, the T_2_WI and DCE images were referenced. The ROIs should cover the whole tumor as much as possible on the largest area of the tumor. Then, all texture features of the tumor were automatically extracted, with a total of 1,050.

### 2.4. Pathological Examination

Postoperative Ki-67 testing was performed by two professional pathologists with more than eight years of pathological diagnosis experience. Immunohistochemical staining was performed by the streptavidin-peroxidase (SP) method. When there were clear brownish yellow granules in the cytoplasm of tumor cells and the staining intensity was higher than that of the nonspecific staining background, Ki-67 expression was determined to be positive. Ten fields were randomly selected under 200x field of vision, and the average tumor positive percentage of each field was used as the proliferation index (PI). The tumor lesions were divided into two groups according to the Ki-67 PI: the Ki-67 PI <50% group and the Ki-67 PI ≥50% group.

### 2.5. Statistical Analysis

A commercial software (SPSS 22.0, IBM Corporation, Armonk NY, USA) was used to analyze the data. Data that had a normal distribution were expressed as mean ± standard deviation (SD), while nonnormally distributed data were expressed in median (M). Independent sample *t*-test was used to conform to the normal distribution, while Mann–Whitney *U*-test in the nonparametric rank sum test was used to conform to the nonnormal distribution. *P* < 0.05 was considered as a statistically significant. Texture features with statistical significance between the two groups were analyzed by univariate and multivariate logistic regression and cross-validation to select the best texture features. Intraclass correlation coefficients (ICCs) were used to evaluate the interobserver agreement in the measurement of texture features (ICC >0.75 was indicative of almost perfect agreement). For parameters with *P* < 0.05, the ROC was drawn, and the area under the curve (AUC) was analyzed to evaluate the accuracy, sensitivity, and specificity. All cases were randomly divided into the training set and the testing set at a ratio of 7 : 3 to verify the accuracy, sensitivity, and specificity of the predictive model and calculate its AUC value.

## 3. Results

Among the 70 patients, 16 patients had Ki-67 PI <50% and 54 patients had Ki-67 PI ≥50%. The tumor diameter revealed a significant difference in these two groups, in which this was larger in the Ki-67 PI ≥50% group than in the Ki-67 PI <50% group. However, there was no significant difference in tumor histological grade and patient age between the two groups (Table 1).

**Figure 1:**
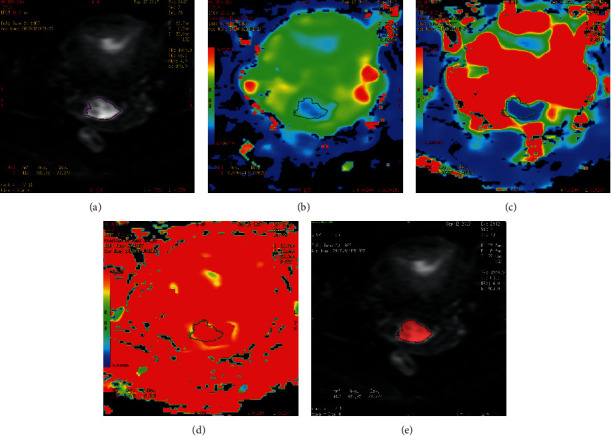
Example of manually drawing ROI for CSCC (Ki-67 PI, 30%). For IVIM-DWI measurement, on panel A (*b* = 800 s/mm^2^), two radiologists drew ROI three times to get the values on the maps of *D*, *D*^*∗*^, and *f*, respectively (panels B–D). For texture analysis, on panel E, the radiologists drew the ROI at the maximum area of the lesion in IVIM-DWI. ROI, region of interest; IVIM-DWI, intravoxel incoherent motion diffusion weighted imaging; PI, proliferation index; *D*, diffusion coefficient; *D*^*∗*^, pseudodiffusion coefficient; *f*, microvascular volume fraction.

**Figure 2:**
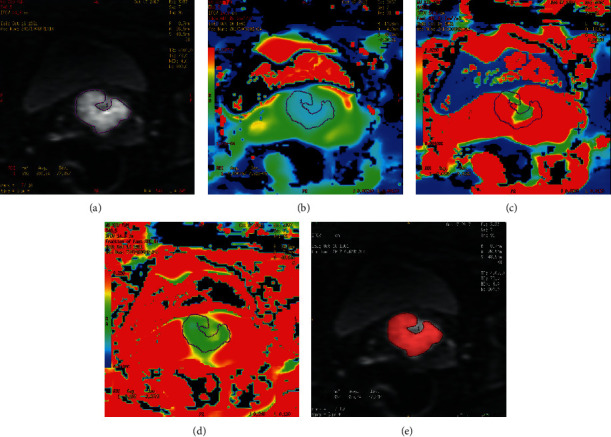
Example of manually drawing ROI for CSCC (Ki-67 PI, 60%). For IVIM-DWI measurement, on panel A (*b* = 800 s/mm^2^), two radiologists drew ROI three times to get the values on the maps of *D*, *D*^*∗*^, and *f*, respectively (panels B–D). For texture analysis, on panel E, the radiologists drew the ROI at the maximum area of the lesion in IVIM-DWI. ROI, region of interest; IVIM-DWI, intravoxel incoherent motion diffusion weighted imaging; PI, proliferation index; *D*, diffusion coefficient; *D*^*∗*^, pseudodiffusion coefficient; *f*, microvascular volume fraction.

The pseudocolor image of parameters obtained by the postprocessing software after the IVIM-DWI scanning is shown in Figures 1 and 2. The *D* value was lower in the Ki-67 PI ≥50% group than in the Ki-67 PI <50% group (*P* < 0.05). There was no significant difference in the *D*^*∗*^ and *f* value between the two groups (Table 2).

**Figure 3:**
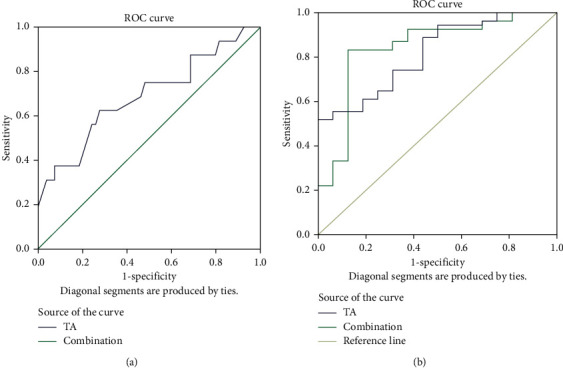
ROC curves of IVIM-DWI *D* (panel (A), overall TA, and IVIM-DWI combined with TA (panel B) for distinguishing Ki-67 PI ≥50% from Ki-67 PI <50% of CSCC. IVIM-DWI, intravoxel incoherent motion diffusion weighted imaging; TA, texture analysis; ROC, receiver operating characteristic; CSCC, cervical squamous cell carcinoma; PI, proliferation index.

In the present study, 1,050 texture features of CSCC based on the IVIM-DWI sequence were obtained. Among these, 37 parameters were statistically different between the two groups. Through univariate and multivariate logistic regression, three texture features were obtained as filtering-independent discriminant features, and the final prediction model was constructed. Finally, a cross-validation was performed to prove that TA is valuable for distinguishing one group from the other, and the results are not due to overfitting (Table 3). The results showed that there was a good interobserver agreement between the two radiologists for the texture features, with ICCs ranging from 0.77–0.97 (Table 4). The ROC curves of IVIM-DWI, TA, and IVIM-DWI combined with TA models are illustrated in Figure 3, and the corresponding values of AUC, accuracy, specificity, and sensitivity are listed in Table 5. The AUC for predicting Ki-67 PI ≥50% through the model was 0.816, and the combination of IVIM-DWI and TA was 0.834. The accuracy, specificity, and sensitivity of the testing set were 0.91, 0.80, and 0.94, respectively, through modeling analysis. When the ROC was used to evaluate the diagnostic efficacy of the prediction model, the AUC of the testing set was 0.899 (Table 6).

## 4. Discussion

In recent years, studies on tumor biomarkers have continuously increased, aiming to improve the diagnosis, treatment methods, and the quality of life and survival rate of tumor patients. Among these, Ki-67 PI was considered as a kind of nuclear protein related to cell proliferation, which is encoded by the MKI67 gene, and expressed in all stages of the cell cycle, except in the G0 phase. The Ki-67 PI in tumors is not only closely correlated to tumor proliferation and invasiveness but also closely correlated with the efficacy of neoadjuvant radiotherapy and chemotherapy on tumors and the prognosis of patients [5]. At present, the biological value of Ki-67 expression in cervical cancer has been certified, which included improving the accuracy, sensitivity, and specificity for cervical cancer screening, improving the repeatability of histopathological diagnosis, monitoring high-risk patients, and following up after treatment [1]. However, as influenced by the limitations of biopsy sampling and heterogeneity of tumors, the noninvasive preoperative evaluation of Ki-67 PI has important clinical significance in predicting the efficacy and judging the prognosis of patients.

IVIM-DWI is a technique that uses a double exponential model to separate water molecule diffusion movement and blood flow microperfusion, which can well reflect the difference in cell density and blood microcirculation perfusion in tumor tissues, and better reveal the diffusion information of biological tissues [6]. The parameters include the following: diffusion coefficient (*D*) represents the diffusion movement of pure water molecules in tissues, equivalent to the density of tumor cells; pseudodiffusion coefficient (*D*^*∗*^) reflects the microcirculation blood perfusion of capillaries, mainly the blood flow velocity in the microcirculation; microvascular volume fraction (*f*) represents the volume ratio of intravoxel microcirculation perfusion to the total diffusion effect, generally referring to the vascular capacity in the microcirculation.

Texture analysis is a postprocessing method for extracting information by quantifying the spatial distribution of pixels or voxels with different gray intensities and counting the variables, that is, calculating and extracting texture features based on the texture matrix of images. This is one of the more commonly used methods in radiomics research [7, 8]. The first-order feature describes the gray distribution of individual pixel values in ROIs. The second-order feature calculates the local texture features represented by two adjacent pixels. The common methods include the gray-level co-occurrence matrix (GLCM) and gray-level run length matrix (GLRLM). The higher order feature analyzes the local image information. Fanet al. [3] and Tagliafico et al. [9] reported that texture features extracted from tumors were significantly correlated with the proliferative status of Ki-67. Nam et al. [10] found that the Ki-67 expression in tumors was closely correlated with the determination of whether the tumors were at low risk based on the texture features obtained by DCE-MRI.

In the present study, the maximum diameter of CSCC was smaller in the Ki-67 PI <50% group than in the Ki-67 PI ≥50% group, and the difference between these two groups was statistically significant. This suggests that the higher the Ki-67 PI in the tumor, the more vigorous the tumor proliferation, and the larger the tumor diameter. This is consistent with the results reported by Yan et al. [11] on Ki-67 PI in breast cancer. In the present study, there was no significant difference in age between the two groups, indicating that the Ki-67 PI is regulated by tumors and that there is no clear relationship with age. In addition, the difference in the histological grade of CSCC between the two groups was not statistically significant, which is inconsistent with the results of previous studies conducted by Yan et al. [11] and Shin and Kim [12]. That is, Ki-67 PI is positively correlated with histological grade. The reason may be correlated with the small sample size of the present study and the large difference in number between the two groups. In general, the higher the histological grade of the tumor, the lower the degree of differentiation, and the more vigorous the proliferation of the tumor cells. Furthermore, the higher the Ki-67 PI becomes, the more the Ki-67 PI would be positively correlated with the histological grade of the tumor.

In the present study, it was found that the *D* value was lower in the Ki-67 PI ≥50% group than in the low expression group. That is, the Ki-67 PI was positively correlated with the density of tumor cells. This could be used as a simple and rapid tool to evaluate the proportion of tumor cell proliferation, which is consistent with the report of Alexey et al. [13]. The *D*^*∗*^ value was lower in the Ki-67 PI ≥50% group than in the Ki-67 PI <50% group, which shows that the density of tumor cells increased when Ki-67 PI ≥50% and the extracellular space decreased. In addition, the microvasculature in the tumor was immature, which was often accompanied by tumor thrombus formation, leading to the slowing of blood flow in the microcirculation. These findings agree with results reported by Xiao et al. [14], which may be correlated with the relatively poor measurement reproducibility of low signal-to-noise ratio (SNR) and *D*^*∗*^ values. The *f* value associated with tumor microcirculation blood volume was higher in the Ki-67 PI ≥50% group than in the low expression group. This was in line with that in previous studies, which demonstrated that Ki-67 PI is positively correlated with the tumor stage. The higher the tumor stage, the higher the malignancy, and the richer the microangiogenesis in the tumor. Hence, the blood volume of tumor microcirculation was relatively more consistent. At present, in the study of glioma, breast cancer, and gastric cancer, it has been generally accepted that the ADC value is negatively correlated with Ki-67 PI. Hence, it was speculated that the ADC value can be used to predict the proliferation of tumors. The ADC value is affected by the elevation of microcirculatory blood perfusion and cannot often truly reflect the spread of tumor tissues, while the *D* value eliminates the influence of microcirculatory blood perfusion in tumors, and the results are more reliable. By drawing the ROC curve and calculating the AUC of parameters with statistical significance difference between the two groups for IVIM-DWI and taking AUC >0.5 as the criterion, it was concluded that the *D* value has high diagnostic efficacy, with a sensitivity of 71.4% and a specificity of 63.5%, for diagnosis based on the 0.516 × 10^−3^ mm^2^/s cutoff value. Therefore, the present study speculated that the *D* value could accurately and noninvasively predict the Ki-67 PI in CSCC before surgery, providing simple and objective imaging help for clinicians in the diagnosis and treatment of the patient's prognosis.

Three texture features were finally selected by the multivariate logistic regression in the present study. Among these, GLRLM_Long run high gray-level emphasis (LRHGLE) was used to measure the joint distribution of long run lengths with higher gray-level values. Firstorder_Interquartile Range = P75–P25. Here, P25 and P75 were the 25^th^ and 75^th^ percentiles of the image array, respectively. GLCM_Informational Measure of Correlation (IMC) 1 assessed the correlation between the probability distributions of I and J (quantifying the complexity of the texture). The prediction accuracy, specificity, and sensitivity of the testing set was 77.7%, 80.2%, and 69.6%, respectively, based on the above three texture features modeling analysis. When the ROC was used to evaluate the diagnostic efficiency of the prediction model, the AUC for the testing set was 0.816. It can be explained that the texture analysis based on IVIM-DWI can be used as a new imaging method to predict the proliferation of tumors and guide the treatment and prognosis of CSCC.

Finally, the combined *D* value and three texture features predicted that the AUC value of Ki-67 PI ≥50% could reach up to 0.834, and the accuracy, sensitivity, and specificity were 86.7%, 74.1%, and 88.7%, respectively. The AUC value, accuracy, and specificity were higher when compared to those in the single method, and the sensitivity was relatively higher. The above analysis results of the present study also show that IVIM-DWI combined with TA on IVIM-DWI has high accuracy, sensitivity, and specificity in predicting Ki-67 PI in CSCC and has higher diagnostic value.

There have some limitations in the present study. First, the present study was retrospective in nature and has a small sample size, which made the robustness of the study results poor. Second, it was difficult to eliminate the error of delineating the edge of the mass and the real situation layer by layer when acquiring texture features. Hence, this also has a certain impact on the research results. Finally, the surgical specimens detected for Ki-67 PI did not correspond to the radiographic images one-to-one. The present study was mainly based on the IVIM-DWI images, and the largest two-dimensional level of the tumor was used to obtain the texture features. Ng et al. [15] have reported sufficient research results using two-dimensional texture analysis, although whole-tumor analysis provides more representative information on tumor heterogeneity. In the future, the investigators would continue the present research, expand the sample size, gradually combine with automatic sketching technology instead of manual sketching ROI, and improve the robustness of the study.

## 5. Conclusions

IVIM-DWI combined with TA based on IVIM-DWI can noninvasively predict the Ki-67 PI in CSCC before surgery, which has important clinical value in detecting high-risk patients, predicting the therapeutic effect, and judging the prognosis of patients. Furthermore, this provides more accurate and objective imaging markers for the clinical diagnosis and treatment of CSCC.

## Figures and Tables

**Table 1:** Comparisons of clinicopathologic characteristics between the high and low Ki-67 PI groups.

	Age (years)	Diameter (cm)	Histological grade [(%)]		
			I	II	III
Ki-67 PI <50% group	49.06 ± 8.10	2.96 ± 1.35	3 (18.75)	3 (18.75)	10 (62.5)
Ki-67 PI ≥50% group	51	3.5	9 (16.67)	17 (31.48)	28 (51.85)
Total/*Z* value	0.693	2.003	1.007		
*P* value	0.488	0.045	0.684		

PI, proliferation index.

**Table 2:** Statistical results of the IVIM-DWI parameters of the high and low Ki-67 PI groups in CSCC.

	*D* (×10^−3^ mm^2^/s)	*D* ^*∗*^ (×10^−3^ mm^2^/s)	*f*
Ki-67 PI <50% group	0.711 ± 0.142	22.670	0.160
Ki-67 PI ≥50% group	0.619 ± 0.105	10.645	0.240
*Z*/*t* value	2.840	−1.741	−1.147
*P* value	0.006	0.082	0.251

PI, proliferation index; IVIM-DWI, intravoxel incoherent motion diffusion weighted imaging; CSCC, cervical squamous cell carcinoma; *D*, diffusion coefficient; *D*∗, pseudodiffusion coefficient; *f*, microvascular volume fraction.

**Table 3:** Statistical results of the univariate analysis and multivariate logistic regression analysis of the texture features of the high and low Ki-67 PI groups in CSCC.

Variable	Ki-67 PI <50% group	Ki-67 PI ≥50% group	Univariate analysis (*p*)	Multivariate logistic regression analysis		
				OR	95% CI	*P*
Wavelet_HHL_GLRLM_LRHGLE	14.591	21.100	0.005	15.93	1.11–228.85	0.042
Wavelet_HLH_GLCM_DE	0.761	0.804	0.011			
Wavelet_LHH_GLSZM_LGLZE	0.490	0.419	0.007			
lbp_3D_k_firstorder_IR	0.615	0.445	0.017	0.46	0.20–1.06	0.047
Wavelet_HLH_GLCM_IMC1	−0.169	−0.136	0.017	1.98	1.05–3.71	0.034
Wavelet_HHH_GLRLM_LRHGLE	14.412	2.534	0.019			
lbp_3D_k_firstorder_skewness	0.001	1.000	0.027			
Wavelet_LHL_GLCM_IDN	1.726	2.143	0.027			

PI, proliferation index; IVIM-DWI, intravoxel incoherent motion diffusion weighted imaging; CSCC, cervical squamous cell carcinoma; CI, confidence interval; GLCM, gray-level co-occurrence matrix; GLRLM, gray-level run length matrix; GLSZM, gray-level size zone matrix; IMC, informational measure of correlation; LRHGLE, long run high gray-level emphasis; DE, difference entropy; LGLZE, low gray-level zone emphasis; IR, interquartile range; IDN, inverse difference normalized.

**Table 4:** Statistical results of the ICC analysis of texture features with statistically significant differences between the high and low Ki-67 PI groups in CSCC.

Texture features	ICC	95% CI
Wavelet_HHL_GLRLM_LRHGLE	0.829	0.578–0.937
Wavelet_HLH_GLCM_DE	0.968	0.910–0.989
Wavelet_LHH_GLSZM_LGLZE	0.820	0.567–0.933
lbp_3D_k_firstorder_IR	0.894	0.723–0.962
Wavelet_HLH_GLCM_IMC1	0.849	0.624–0.944
Wavelet_HHH_GLRLM_LRHGLE	0.827	0.570–0.936
lbp_3D_k_first order_skewness	0.767	0.448–0.912
Wavelet_LHL_GLCM_IDN	0.781	0.488–0.917

ICC, intraclass correlation coefficient; CI, confidence interval; GLCM, gray-level co-occurrence matrix; GLRLM, gray-level run length matrix; GLSZM, gray-level size zone matrix; IMC, informational measure of correlation; LRHGLE, long run high gray-level emphasis; DE, difference entropy; LGLZE, low gray-level zone emphasis; IR, interquartile range; IDN, inverse difference normalized.

**Table 5:** The ROC analysis for each individual feature, overall IVIM-DWI, overall TA, and IVIM-DWI combined with TA.

Parameters	AUC	95% CI	Accuracy (%)	Sensitivity (%)	Specificity (%)
D (×10^−3^mm^2^/s）	0.689	0.528–0.849	67.5	62.5	72.2
Wavelet_LHH_GLSZM_LGLZE	0.725	0.582–0.867	68.6	64.8	81.3
lbp_3D_k_firstorder_IR	0.697	0.555–0.838	75.7	81.5	56.3
Wavelet_HLH_GLCM_IMC1	0.697	0.532–0.862	78.6	85.2	56.3
Overall TA	0.816	0.708–0.924	75.3	77.8	87.5
D combined with TA	0.834	0.715–0.953	86.7	74.1	88.7

IVIM-DWI, intravoxel incoherent motion diffusion weighted imaging; TA, texture analysis; ROC, receiver operating characteristic; AUC, area under curve; CI, confidence interval; D, diffusion coefficient; GLCM, gray-level co-occurrence matrix; GLSZM, gray-level size zone matrix; IMC, informational measure of correlation; LGLZE, low gray-level zone emphasis; IR, interquartile range.

**Table 6:** The training set and testing set verify the predictive model's diagnostic efficiency.

	Ki-67 PI ＜50% group (*n*)	Ki-67 PI ≥50% group (*n*)	Accuracy (%)	Specificity (%)	Sensitivity (%)	*F*-value	AUC
Training set	11	37	87.5	72.7	91.9	0.94	0.907
Testing set	5	17	90.9	80.0	94.1	0.94	0.899

PI, proliferation index; AUC, area under curve.

## Data Availability

The raw data supporting the conclusions of this article will be made available by the authors, without undue reservation.

## Supplementary Materials

Supplementary Materials Supplementary Table 1: introducing the texture features. Supplementary Figure 1: the process of patient selection.
