# Supplementary material for: Predictive Ki-67 Proliferation Index of Cervical Squamous Cell Carcinoma Based on IVIM-DWI Combined with Texture Features
Source: Contrast Media Mol Imaging. 2021 Jan 14;2021:8873065. doi: 10.1155/2021/8873065 (PMC7826202; doi:10.1155/2021/8873065)
Supplement: Supplementary Materials — Supplementary Table 1: introducing the texture features. Supplementary Figure 1: the process of patient selection. [file 8873065.f1.zip › 8873065.f1/Supplementary material.pdf]

Supplementary material

Table S1 Texture features in this study

Method	Features
First order	Energy, Total Energy, Entropy, Minimum, 10th Percentile, 90th Percentile, Maximum, Mean, Median, Interquartile Range (IR), Range, Mean Absolute Deviation (MAD), Robust Mean Absolute Deviation (RMAD), Root Mean Squared (RMS), Skewness, Kurtosis, Variance, Uniformity
GLCM	Autocorrelation, Joint Average, Cluster Prominence, Cluster Shade, Cluster Tendency, Contrast, Correlation, Difference Average, Difference Entropy, Difference Variance, Joint Energy, Joint Entropy, Informational Measure of Correlation (IMC) 1, Informational Measure of Correlation (IMC) 2, Inverse Difference Moment (IDM), Maximal Correlation Coefficient (MCC), Inverse Difference Moment Normalized (IDMN), Inverse Difference (ID), Inverse Difference Normalized (IDN), Inverse Variance, Maximum Probability, Sum Average, Sum Entropy, Sum of Squares
GLRLM	Short Run Emphasis (SRE), Long Run Emphasis (LRE), Gray Level Non-Uniformity (GLN), Gray Level Non-Uniformity Normalized (GLNN), Run Length Non-Uniformity (RLN), Run Length Non-Uniformity Normalized (RLNN), Run Percentage (RP), Gray Level Variance (GLV), Run Variance (RV), Run Entropy (RE), Low Gray Level Run Emphasis (LGLRE), High Gray Level Run Emphasis (HGLRE), Short Run Low Gray Level Emphasis (SRLGLE), Short Run High Gray Level Emphasis (SRHGLE), Long Run Low Gray Level Emphasis (LRLGLE), Long Run High Gray Level Emphasis (LRHGLE)
GLSZM	Small Area Emphasis (SAE), Large Area Emphasis (LAE), Gray Level Non-Uniformity (GLN), Gray Level Non-Uniformity Normalized (GLNN), Size-Zone Non-Uniformity (SZN), Size-Zone Non-Uniformity Normalized (SZNN), Zone Percentage (ZP), Gray Level Variance (GLV), Zone Variance (ZV), Zone Entropy (ZE), Low Gray Level Zone Emphasis (LGLZE), High Gray Level Zone Emphasis (HGLZE), Small Area Low Gray Level Emphasis (SALGLE), Large Area Low Gray Level Emphasis (LALGLE), Large Area High Gray Level Emphasis (LAHGLE)

GLCM, gray level cooccurrence matrix; GLRLM, gray level run length matrix; GLSZM, gray level size zone matrix
