# Supplementary figures and images for: Predictive Ki-67 Proliferation Index of Cervical Squamous Cell Carcinoma Based on IVIM-DWI Combined with Texture Features
Source: Contrast Media Mol Imaging. 2021 Jan 14;2021:8873065. doi: 10.1155/2021/8873065 (PMC7826202; doi:10.1155/2021/8873065)

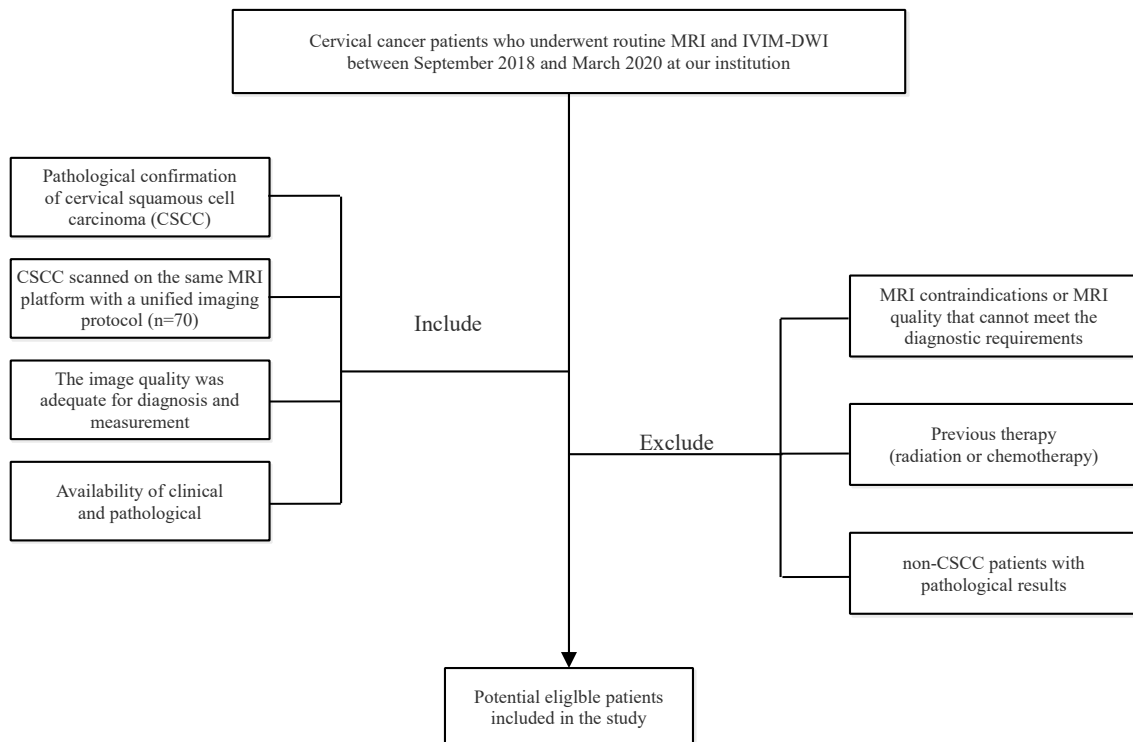

Supplement: Supplementary Materials — Supplementary Table 1: introducing the texture features. Supplementary Figure 1: the process of patient selection. [file 8873065.f1.zip › 8873065.f1/Figure S1.pdf]
